# Anti-Inflammatory Effects of Metabolites from Antarctic Fungal Strain *Pleosporales* sp. SF-7343 in HaCaT Human Keratinocytes

**DOI:** 10.3390/ijms22189674

**Published:** 2021-09-07

**Authors:** Linsha Dong, Hye Jin Kim, Thao Quyen Cao, Zhiming Liu, Hwan Lee, Wonmin Ko, Youn-Chul Kim, Jae Hak Sohn, Tai Kyoung Kim, Joung Han Yim, Dong-Sung Lee, Hyuncheol Oh

**Affiliations:** 1 College of Pharmacy, Chosun University, Dong-gu, Gwangju 61452, Korea; donglinsha011@163.com (L.D.); lzmqust@126.com (Z.L.); ghksdldi123@hanmail.net (H.L.); rabis815@naver.com (W.K.); 2 Institute of Pharmaceutical Research and Development, College of Pharmacy, Wonkwang University, Iksan 54538, Korea; mn1003@naver.com (H.J.K.); quyen.cao.thao@gmail.com (T.Q.C.); yckim@wku.ac.kr (Y.-C.K.); 3 Hanbang Cardio-Renal Syndrome Research Center, Wonkwang University, Iksan 54538, Korea; 4 College of Medical and Life Sciences, Silla University, Busan 46958, Korea; jhsohn@silla.ac.kr; 5 Division of Polar Life Sciences, Korea Polar Research Institute, Incheon 21990, Korea; tkkim@kopri.re.kr (T.K.K.); jhyim@kopri.re.kr (J.H.Y.)

**Keywords:** Antarctic fungi, inflammation, ICAM-1, NF-κB, HO-1, HaCaT

## Abstract

Chemical investigation of the Antarctic fungi *Pleosporales* sp. SF-7343 revealed four known secondary fungal metabolites: alternate C (**1**), altenusin (**2**), alternariol (**3**), and altenuene (**4**). The compound structures were identified primarily by NMR and MS analyses. Atopic dermatitis, an inflammatory disease, is driven by the abnormal activation of T helper (Th) 2 cells and barrier dysfunction. We attempted to identify the anti-inflammatory components of SF-7343. Initial screening showed that compounds **1** and **3** inhibited the secretion of interleukin-8 and -6 in tumor necrosis factor-α/interferon-γ-treated HaCaT cells, and these compounds also showed inhibitory effects on CCL5 and CCL22. Compounds **1** and **3** also downregulated the protein expression levels of intercellular adhesion molecule-1 and upregulated the expression of filaggrin and involcurin. The mechanism study results showed that compounds **1** and **3** inhibited nuclear translocation of nuclear factor-kappa B p65 and the phosphorylation of STAT1 and STAT3. Compound **1**, but not compound **3**, significantly promoted the expression of heme oxygenase (HO)-1. The effects of compound **1** were partly reversed by co-treatment with a HO-1 inhibitor, tin protoporphyrin IX. Taken together, this study demonstrates the potential value of Antarctic fungal strain SF-7343 isolates as a bioresource for bioactive compounds to prevent skin inflammation.

## 1. Introduction

Atopic dermatitis (AD) is a chronic inflammatory skin disease, and the causes of this disease are complex and multifactorial. The prevalence of AD is quite high, at 20% in children and 10% in adults [1]. Common symptoms of AD include dry and itchy skin, eczema, and redness. Furthermore, AD is also associated with other atopic disorders, skin ulcers, and sleep disorders, and leads to a lower quality of life [2]. From an immunological perspective, AD is a helper (Th)2 cell-mediated skin disease. This is caused by an imbalance between the levels of Th1 and Th2 cells [3]. Some inflammatory cytokines and chemokines that are secreted by Th2 cells can affect skin cells, such as keratinocytes and mast cells. When mast cells are activated, histamine, along with many other cytokines and chemokines, are released, which promote the infiltration of immune cells into inflammatory lesions [4]. In the lesion site, a further inflammation-related reduction in skin barrier function, enhanced irritability and scratching-related stimuli deteriorate eczema, leading to a vicious cycle of inflammation. Furthermore, it also induces the overproduction of immunoglobulin E (lgE), eventually accelerating the progression of AD [5].

Keratinocytes are the most abundant cells in the epidermis and represent the first line of the host defense system. These cells form the physical skin barrier by sensing pathogens via innate immune receptors, initiating anti-microbial responses, and producing various cytokines, chemokines, and anti-microbial peptides [6]. In AD-damaged epidermal barrier, the penetration of potential allergens and pathogens activates the keratinocytes. Among the dysregulation of immune responses in AD, activated keratinocytes play an important role in several biological processes that contribute to the pathogenesis of AD [7].

Due to the unique location and climate of Antarctica, the microorganisms of Antarctica are highly adapted to and can endure extreme conditions [8]. Fungi play an important role in the search for structurally unique secondary metabolites. Many studies have shown that Antarctic fungi produce bioactive metabolites [9]. Secondary metabolites derived from Antarctic fungi can be categorized into alkaloids, terpenoids, polyketides, peptides, and sterols. Thus, Antarctic fungi are attractive sources for the isolation of new secondary metabolites with novel structures [10,11]. In our research on bioactive secondary metabolites from Antarctic fungi, we investigated the chemistry of the EtOAc extract obtained from the culture of the fungal strain *Pleosporales* sp. SF-7343, which led to the isolation of four compounds such as alternate C (compound **1**), alternariol (compound **2**), alternariol (compound **3**), and altenuene (compound **4**). In a previous study, alternariol are only reported to suppress LPS-induced inflammation in THP-1-derived macrophages [12]. However, there have been no reports yet that alternate C, alternariol and altenuene exert anti-inflammatory effects. This is the first report on the regulatory effects of skin inflammatory factors by using compounds **1**–**4** in HaCaT cells. This study describes the isolation and structural elucidation of these four compounds form metabolites from the Antarctic fungal strain *Pleosporales* sp. SF-7343, along with their anti-inflammatory effects in tumor necrosis factor-α/interferon-γ (TNF-α/IFN-γ)-stimulated HaCaT cells.

## 2. Results

### 2.1. Structural Determination of Isolated Metabolites

The dried extract of the cultured fungal strain *Pleosporales* sp. SF-7343 was subjected to multiple chromatographic steps to obtain alternate C (**1**) [13], altenusin (**2**) [14], alternariol (**3**) [15], and altenuene (**4**) [16]. Their structures (Figure 1) were identified based on 1D and 2D NMR, MS analyses (Figures S1–S20), and by comparison with reported data in the literature.

### 2.2. Effects of Compounds ***1***, ***2***, ***3***, and ***4*** on Cell Viability

**Figure 2:**
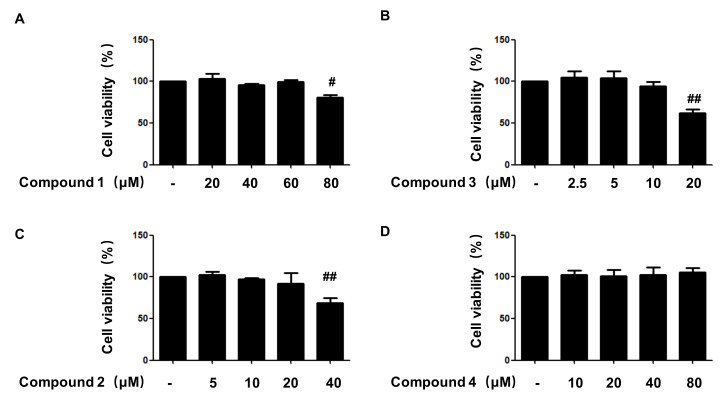
Cytotoxicity of the compounds **1** (**A**), **2** (**C**), **3** (**B**), and **4** (**D**)used in this study. Cytotoxicity was evaluated in cells treated for 24 h with indicated concentration of 4 compounds. Data are presented as the mean ± SD values of 3 independent experiments. ^#^ *p* < 0.05, ^##^ *p* < 0.01, vs. control.

We evaluated the cytotoxicity of compounds **1**, **2**, **3**, and **4** on HaCaT cells using the MTT assay. The cell viability did not alter following the addition of 80 μM concentrations of compounds **4**, for 24 h. However, compound **1** at 80 μM, compound **2** at 40 μM, and compound **3** at 20 μM demonstrated cell toxicity in HaCaT cells. Therefore, based on the results of Figure 2, further experiments were conducted using each compound in the concentration range of non-toxicity such as compound **1** (20–60 μM), **2** (5–20 μM), **3** (2.5–10 μM), and **4** (20–80 μM).

### 2.3. Effects of Compounds ***1***, ***2***, ***3***, and ***4*** on the Secretion of IL-6 in TNF-α/IFN-γ-Stimulated HaCaT Cells

**Figure 3:**
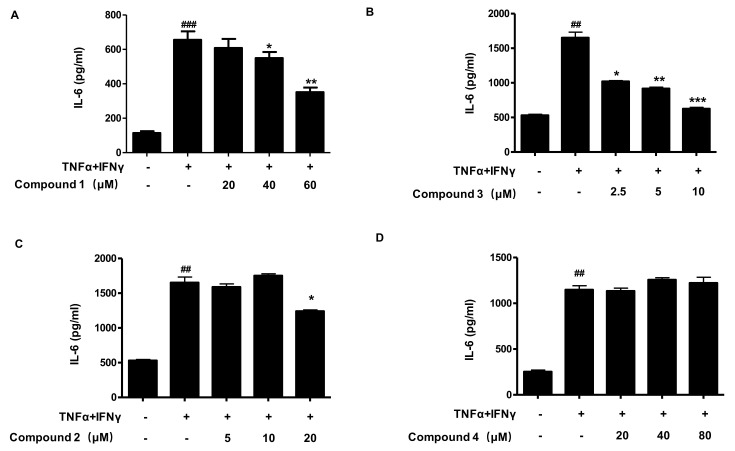
Effects of compounds **1**, **2**, **3**, and **4** on the secretion of IL-6 in TNF-α/IFN-γ-stimulated HaCaT cells (**A**–**D**). The secretion of IL-6 was measured by using the culture supernatant of TNF-α/IFN-γ-stimulated HaCaT cells. The cells were pretreated with the indicated concentrations of compound **1**, **2**, **3**, and **4** for 3 h (+) or not (-), and then stimulated with TNF-α/IFN-γ (+) for 24 h. Data are represented as the mean ± SD of three independent experiments. ^##^ *p* < 0.01, ^###^ *p* < 0.001 vs. control. * *p* < 0.05, ** *p* < 0.01, *** *p* < 0.001 vs. TNF-α/IFN-γ-treated group.

To determine the anti-inflammatory effects of compounds **1**, **2**, **3**, and **4** in TNF-α/IFN-γ-stimulated HaCaT cells, we first analyzed the secretion of IL-6. As shown in Figure 3, compounds **1** and **3** significantly inhibited the secretion of IL-6. However, compound **4** had no significant effect on IL-6 secretion and compound **2** showed the inhibitory effect of IL-6 secretion at only high concentration. Among the 4 compounds, compounds **1** and **3** showed the most remarkable effect in inhibiting the secretion of IL-6 in HaCaT cells. Therefore, the most effective two compounds were selected for further experiments.

### 2.4. Effects of Compounds ***1*** and ***3*** on IL-8, MDC, and RANTES Secretion in TNF-α/IF-γ-Stimulated HaCaT Cells

**Figure 4:**
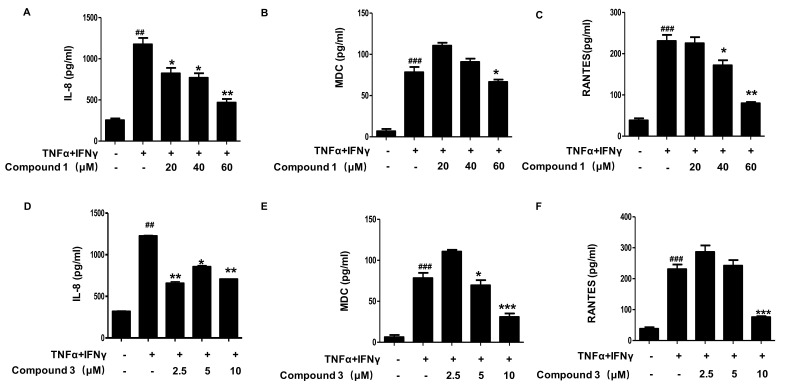
Effects of compounds **1** and **3** on IL-8 (**A**,**D**), MDC (**B**,**E**), and RANTES (**C**,**F**) secretion in TNF-α/IFN-γ-stimulated HaCaT cellsThe levels of IL-8, MDC, and RANTES were measured using the culture supernatant of TNF-α/IFN-γ-stimulated HaCaT cells. The cells were pretreated with the indicated concentrations of compounds **1** and **3** for 3 h (+) or not (-), and then stimulated with TNF-α/IFN-γ (+) for 24 h. Data are represented as the mean ± SD of three independent experiments., ^##^ *p* < 0.01, ^###^ *p* < 0.001 vs. control. * *p* < 0.05, ** *p* < 0.01, *** *p* < 0.001 vs. TNF-α/IFN-γ-treated group.

The epidermis is not only a physical barrier, but also a chemical and immunological barrier, producing various cytokines and chemokines. Therefore, next, the effects of compounds **1** and **3** on other inflammatory cytokines and chemokines were determined in Figure 4. Levels of IL-8, macrophage-derived chemokine (MDC), and regulated upon activation, normal T cell-expressed and presumably -secreted (RANTES) were determined from the cell culture supernatant using ELISA. In the TNF-α/IFN-γ-stimulated group, the secretion levels of IL-8, MDC, and RANTES were significantly increased compared to the control group, which were dose-dependently reduced by pre-treatment with compounds **1** and **3**.

### 2.5. Effects of Compounds ***1*** and ***3*** on TNF-α/IFN-γ-Induced ICAM-1 Expression in HaCaT Cells

**Figure 5:**
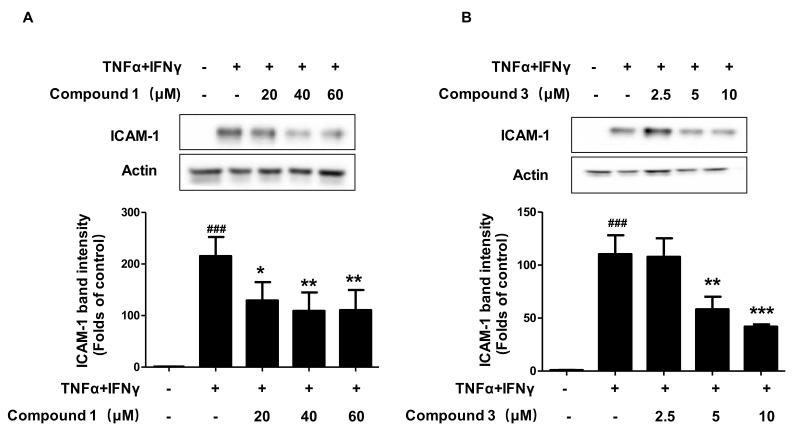
Effects of compounds **1** and **3** on TNF-α/IFN-γinduced ICAM-1 expression in HaCaT cells (**A**,**B**) The expression of ICAM-1 was measured using Western blotting. The cells were pretreated with the indicated concentrations of compounds **1** and **3** for 3 h (+) or not (-), and then stimulated with TNF-α/IFN-γ (+) for 24 h. Data are represented as the mean ± SD of three independent experiments. ^###^ *p* < 0.001 vs. control. * *p* < 0.05, ** *p* < 0.01, *** *p* < 0.001 vs. TNF-α/IFN-γ-treated group.

Under inflammatory conditions, keratinocytes can also express adhesion molecules, such as ICAM-1. The expression of ICAM-1 promotes the adsorption of leukocytes into nearby skin tissues [17]. We examined the effects of compounds **1** and **3** on TNF-α/IFN-γ-induced ICAM-1 expression. As shown in Figure 5, both compounds **1** and **3** exerted significant effects on ICAM-1 expression in a dose-dependent manner.

### 2.6. Effects of Compounds ***1*** and ***3*** on FLG and IVL Expression, in HaCaT Cells

**Figure 6:**
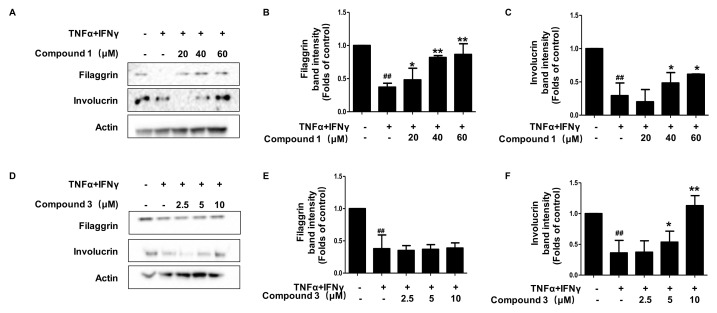
Effects of compounds **1** and **3** on FLG (**A**–**C**) and IVL (**D**–**F**) expression in HaCaT cells. The expressions of FLG and IVL were measured using cell lysis. The cells were pretreated with the indicated concentrations of compounds **1** and **3** for 3 h (+) or not (-), and then stimulated with TNF-α/IFN-γ (+) for 24 h. Data are represented as the mean ± SD of three independent experiments, ^##^ *p* < 0.01 vs. control. * *p* < 0.05, ** *p* < 0.01, vs. TNF-α/IFN-γ-treated group.

Filaggrin (FLG) and involcurin (IVL) are two proteins that encode genes present in the topmost layer of the epidermis, and play a major role in keratinocyte differentiation and skin barrier function [18,19]. In inflammatory keratinocytes, the expression of these two proteins decreases and the skin barrier function is weakened. Our results showed that the treatment of compound **1** restored the decline of FLG and IVL expression compared with the TNF-α/IFN-γ group (Figure 6A–C). As compared to TNF-α/IFN-γ group, compound **3** suppressed the decrease of IVL expression induced by TNF-α/IFN-γ in HaCaT cells (Figure 6D,F). However, compound **3** had no significant effect on FLG expression (Figure 6D,E). Compound **3** probably affects other mechanisms rather than its protective effect on filaggrin.

### 2.7. Effects of Compounds ***1*** and ***3*** on the NF-κB, p-STAT1, and p-STAT3 Signaling Pathways in HaCaT Cells

**Figure 7:**
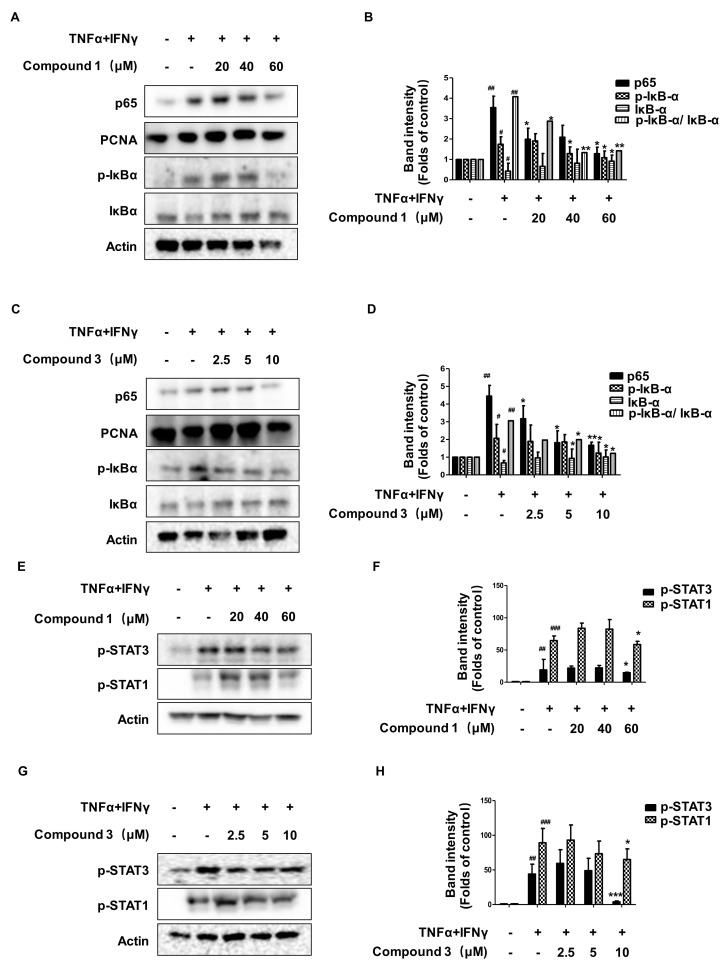
Effects of compounds **1** and **3** on the NF-κB- (**A**–**D**), p-STAT1-, and p-STAT3- (**E**–**H**) signaling pathway in HaCaT cells. The cells were pre-treated with the indicated concentrations of compounds **1** and **3** for 3 h (+) or not (-), and then stimulated with TNF-α/IFN-γ (+) for 15 min. Cytosol and nuclear extracts were isolated, and levels of p65, p-IκBα, and IκBα in fractions were determined by Western blotting. The expression of p-stat3, p-stat1, and total proteins were measured using Western blotting. The bar graphs represent quantitative densities of the bands. Data are represented as the mean ± SD of three independent experiments. ^#^ *p* < 0.05, ^##^ *p* < 0.01, ^###^ *p* < 0.001 vs. control. * *p* < 0.05, ** *p* < 0.01, *** *p* < 0.001 vs. TNF-α/IFN-γ-treated group.

In keratinocytes, TNF-α/IFN-γ co-stimulation leads to the release of cytokines and chemokines, and this process is regulated by the NF-κB-, p-STAT1-, and p-STAT3-signaling pathways [20,21]. As shown in Figure 7A–D, when cells were treated with TNF-α/IFN-γ, the p-IκBα and p65 were significantly increased as compared to the control group. Pretreatment with compounds **1** and **3** decreased the phosphorylation of IκBα and the activation of p65. The ratio of p-IκBα/IκBα also decreased. As shown in Figure 7E–H, compounds **1** and **3** also significantly downregulated the phosphorylation of STAT1 and STAT3. These results suggest that compounds **1** and **3** could regulate the NF-κB-, p-STAT1-, and p-STAT3-signaling pathways in TNF-α/IFN-γ-treated HaCaT cells.

### 2.8. Effects of Compounds ***1*** and ***3*** on HO-1 Expression and Nrf2 Translocation

**Figure 8:**
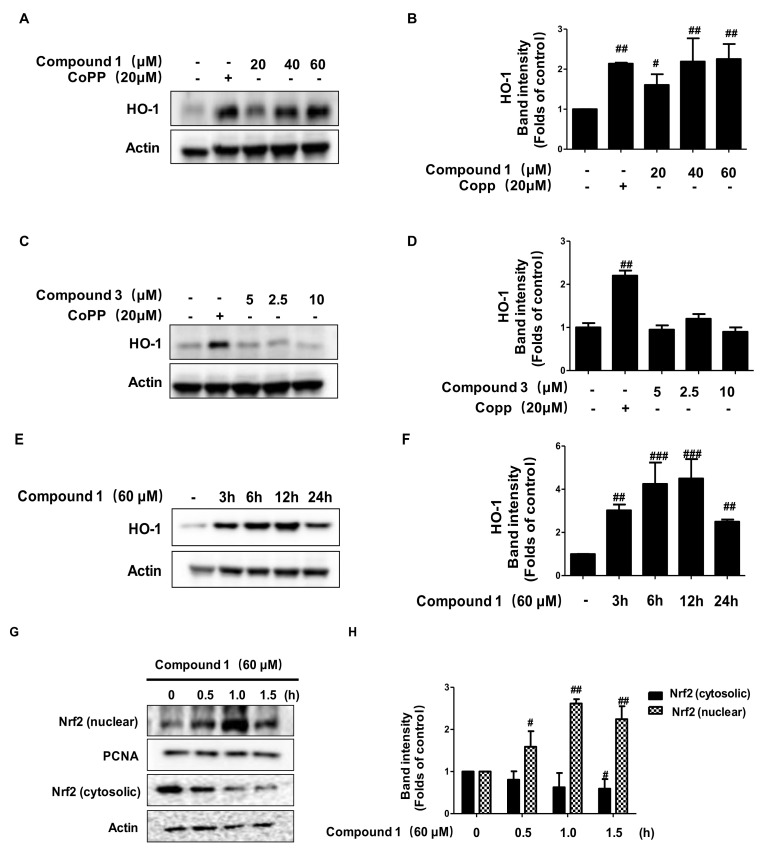
Effects of compounds **1** and **3** on heme oxygenase (HO)-1 expression and Nrf2 translocation. HaCaT cells were incubated for 12 h with the indicated concentration of compound **1** (20–60 μM) and compound **3** (2.5–10 μM) (**A**–**D**). HaCaT cells were incubated with 60 µM of compound **1** for 0–24 h (**E**,**F**). HaCaT cells were treated with 60 µM of compound **1** for 0.5, 1, and 1.5 h (**G**,**H**). The nuclei were fractionated and Western blot analyses for HO-1 and Nrf2 expression were performed. Data are represented as the mean ± SD of three independent experiments. ^#^ *p* < 0.05, ^##^ *p* < 0.01, ^###^ *p* < 0.001 vs. control.

To investigate whether the compounds induced the expression of heme oxygenase (HO)-1, HaCaT cells were treated with the indicated concentrations of compounds **1** (20–60 μM) and **3** (2.5–10 μM), the HO-1 inducer, cobalt protoporphyrin (CoPP), increased HO-1 expression at 20 µM, was used as a positive control. In addition, the results are shown in Figure 8A–D. Compound **1** was able to induce HO-1 expression in a dose- (Figure 8A,B) and time-dependent manner (Figure 8E,F). However, compound **3** failed to induce the expression of HO-1 in HaCaT cells (Figure 8C,D). Next, we examined the effect of compound **1** on the nuclear translocation of Nrf2. As shown in Figure 8G,H, compound **1** significantly promoted the translocation of Nrf2 from the cytosol to the nucleus. These results show that compound **1** could promote the expression of HO-1, and regulated the Nrf2/HO-1 pathway.

### 2.9. Anti-Inflammatory Effects of Compound ***1*** through HO-1 Regulation in TNF-α/IFN-γ-Stimulated HaCaT Cells

**Figure 9:**
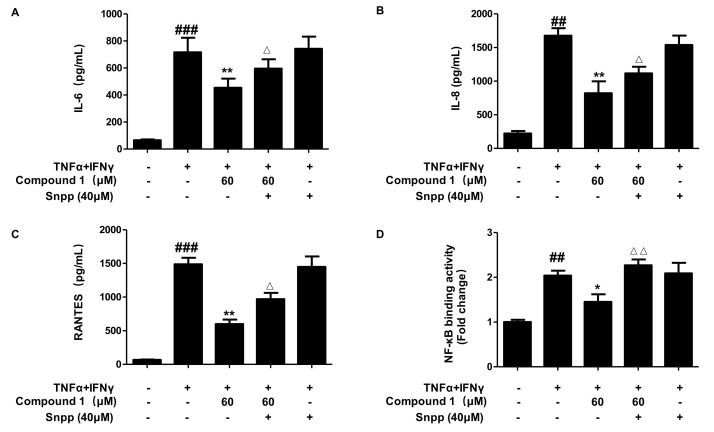
Anti-inflammatory effects of compound 1 through HO-1 regulation (**A**–**D**). HaCaT cells were pre-treated for 3 h with compound **1** (60 μM) or not (-) in the presence (+) or absence (-) of SnPP (40 μM) and stimulated with TNF-α/IFN-γ (+) for 24 h to measure IL-6 (**A**), IL-8 (**B**), and RANTES (**C**) or for 15 min to measure NF-κB binding activity (**D**). The levels of IL-6, IL-8, and RANTES, and NF-κB binding activity were detected by ELISA. Data are represented as the mean ± SD of three independent experiments., ^##^ *p* < 0.01, ^###^ *p* < 0.001 vs. control. * *p* < 0.05, ** *p* < 0.01, vs. TNF-α/IFN-γ-treated group. ^△^ *p* < 0.05, ^△△^ *p* < 0.01, vs. compound **1**+TNF-α/IFN-γ-treated group.

Based on the results that compound **1** could induce the expression of HO-1, we examined whether the anti-inflammatory effects of compound **1** are related to HO-1. We used a selective HO-1 inhibitor, tin protoporphyrin IX (Snpp), and the cells were treated with or without Snpp (40 μM) for 3 h, with or without compound **1** (60 μM), and then treated with TNF-α/IFN-γ for 24 h. The levels of IL-6, IL-8, and RANTES were determined by ELISA. As shown in Figure 9A–C, compound **1** treatment significantly decreased the secretion of IL-6, IL-8, and RANTES induced by TNF-α/IFN-γ. However, as compared to the TNF-α/IFN-γ+compound **1** group, the secretion of IL-6, IL-8, and RANTES was reversed by the compound **1**+Snpp group. Moreover, NF-κB binding activity showed similar results. SnPP treated alone or TNF-α/IFN-γ+SnPP groups did not show effects in cell viability, IL-6, IL-8, RANTES, and NF-κB binding activity. These results suggested that the anti-inflammatory effects of compound **1** were mediated by HO-1 expression.

## 3. Discussion

Many studies have shown that various bioactive compounds exert their anti-inflammatory activities by inducing the expression of HO-1 in inflammatory disease models [22]. HO-1 is the rate-limiting enzyme for the degradation of heme and produces ferrous iron, carbon monoxide, and biliverdin [23]. These three by-products exert various beneficial effects, including anti-inflammatory, antioxidant, and cell protective effects [24], under many pathological conditions. A few studies have shown that HO-1 expression exerts immunomodulatory effects against inflammatory skin diseases, such as AD [22,23]. Nrf2, a transcription factor, binds to Keap1 in the cytoplasm, and when activated, Nrf2 separates from Keap1 and translocates to the nucleus, combines with antioxidant response element (ARE) to upregulate the expression of antioxidant enzymes, including HO-1, and induces cell protection [25,26]. Our results showed that compound **1** could significantly induce the expression of HO-1, while the translocation of Nrf2 from the cytosol to the nucleus was also promoted by alternate C (**1**). The results of pro-inflammatory cytokines and chemokines of HO-1 inhibitor (Snpp) also suggest that the anti-inflammatory effects of alternate C (**1**) are partly mediated by the Nrf2/HO-1 pathway.

Keratinocytes react with pro-inflammatory cytokines such as TNF-α and IFN-γ, which are involved in the expression of many inflammatory mediators during chronic inflammatory skin disorders, such as psoriasis and AD [27,28]. Activated keratinocytes release pro-inflammatory cytokines and chemokines, which play a critical role in the infiltration of inflammatory immune cells, into AD lesions [29,30]. Subsequently, in inflammatory AD lesions, the levels of some pro-inflammatory cytokines and chemokines increase [29]. As a pro-inflammatory cytokine, IL-6 is involved in epidermal hyperplasia in psoriatic epithelium, influences the functioning of dermal inflammatory cells, and induces the differentiation of human Th17 cells [31]. Chemokines, such as IL-8, RANTES, TARC, and MDC, mediate the recruitment of leukocytes to the skin from the blood and play important roles in establishing a microenvironment [32,33]. Targeting pro-inflammatory cytokines and chemokines is a promising strategy for treating different skin diseases that are resistant to existing therapies [34]. We performed in vitro experiments using HaCaT cells to investigate the effects of metabolites isolated from the fungal strain SF-7343 on TNF-α/IFN-γ-stimulated keratinocytes. After stimulation with TNF-α/IFN-γ, HaCaT cells were activated, releasing various cytokines and chemokines. Our study demonstrates that alternate C (**1**) and alternariol (**3**) exert anti-inflammatory effects by reducing the secretion of IL-6, IL-8, MDC, and RANTES.

The activation of STAT and NF-κB is important for increasing AD-related inflammatory gene expression in HaCaT cells [35]. When STAT1, STAT3, and NF-κB are activated by pro-inflammatory cytokines, STAT1, STAT3, and NF-κB are translocated from the cytosol into the nucleus in keratinocytes, where they contribute to the expression of inflammatory mediators [36]. The administration of alternate C (**1**) and alternariol (**3**) to HaCaT cells stimulated with TNF-α and IFN-γ attenuated the increased secretion of inflammatory mediators, and also inhibited STAT1 and STAT3 phosphorylation, IκBα degradation, and NF-κB nuclear translocation. Therefore, we suggest that alternate C (**1**) and alternariol (**3**) have inhibitory effects on the expression of inflammatory cytokines and chemokines via the inhibition of the STAT1, STAT3, and NF-κB signaling pathways.

Increased pro-inflammatory cytokines and chemokines cause alteration of skin barrier proteins, which is one of the main initial factors in the pathogenesis of AD [27]. Some proteins, such as FLG and IVL, which play a major role in keratinocyte differentiation and skin barrier function, resist on the cell surface [37]. Cytokines secreted by keratinocytes and other skin-inhabited cells are involved in cellular communication, thus resulting in an altered barrier function of the skin. For example, cytokines manipulate keratinocyte proliferation and differentiation by regulating gene expression within these cells. Uncontrolled cytokine expression can lead to dysfunction of the epidermal barrier, as seen in diseases such as AD and psoriasis [38,39]. Co-stimulation with TNF-α/IFN-γ for 24 h resulted in the reduction of FLG and IVL, which means that the skin barrier function was partly impaired. Alternate C (**1**) and alternariol (**3**) both upregulated the expression of IVL, but only alternate C (**1**) upregulated the expression of FLG.

## 4. Materials and Methods

### 4.1. General Experimental Procedures

Optical rotations were recorded using a Jasco P-2000 digital polarimeter (JASCO Corp., Tokyo, Japan). Nuclear magnetic resonance (NMR) spectra (1D and 2D) were recorded in DMSO-*d*_6_ (δ_H_/δ_C_ = 2.50/39.52) using a JEOL JNM ECP-400 spectrometer (JEOL Ltd., Tokyo, Japan), and the chemical shifts were referenced relative to the residual solvent peaks. Heteronuclear multiple quantum coherence (HMQC) and heteronuclear multiple bond correlation (HMBC) experiments were optimized for ^1^*J*_CH_ = 140 Hz and ^n^*J*_CH_ = 8 Hz, respectively. HRESIMS data were obtained using an AB Sciex Triple TOF 4600 instrument (AB Sciex Pte. Ltd., Framingham, MA, USA). Thin-layer chromatography (TLC) was performed on Kieselgel 60 F_254_ (1.05715; Merck, Darmstadt, Germany) or RP-18 F_254s_ (Merck) plates. Spots were visualized by spraying plates with 10% aqueous sulfuric acid (H_2_SO_4_) solution, followed by heating. Column chromatography was performed on silica gel (Kieselgel 60, 70–230 mesh and 230–400 mesh, Merck) and YMC octadecyl-functionalized silica gel (C_18_). High-performance liquid chromatography (HPLC) was performed on a preparative-C_18_ column (10 × 250 mm; 5 μm particle size) with a flow rate of 5 mL/min, and the compound was detected by ultraviolet (UV) absorption at 210 and 254 nm.

### 4.2. Fungal Material and Fermentation

The fungal strain SF-7343 was isolated from moss collected from the Marian Cove (62°13′16.5′′ S, 58°46′29.6′′ W) on King George Island, Antarctica, in January 2017. One gram of the sample was ground with a mortar and pestle and mixed with sterile seawater (10 mL). A portion (0.1 mL) of the sample was processed using the spread plate method in PDA medium containing seawater and incubated at 25 °C for 14 d. After sub-culturing the isolates several times, the final pure cultures were selected and stored at −70 °C. The fungal strain SF-7343 was identified based on the ITS gene sequence analysis. A GenBank search with the ITS gene of SF-7343 (GenBank accession number MK785420) indicated that *Pleosporales* sp. Di61-4 (KC514877), *Pleosporales* sp. AM282-P9T2T (KT264548), and *Lophodermium pini-excelsae* (EU520183) were the closest matches, showing sequence identities of 100%, 99.58%, and 99.37%, respectively. Therefore, the fungal strain SF-7343 was characterized as *Pleosporales* sp.

### 4.3. Extraction and Isolation of Metabolites

The fungal strain *Pleosporales* sp. SF-7343 was cultured in 20 Fernbach flasks, each containing 300 mL of PDB medium and 75 g of vermiculite with 3% NaCl. The flasks were individually inoculated with 5 mL of seed cultures of fungal strain and incubated at 25 °C for 14 d. The cultured fungi were directly extracted with EtOAc (20 L), which provided an organic phase, which was then evaporated in vacuo to yield a residue of SF-7343V (3.91 g). The crude extract was fractionated using RP C_18_ flash column chromatography (4.5 × 30 cm), eluting with a stepwise gradient of 20%, 40%, 60%, 80%, and 100% (*v/v)* MeOH in H_2_O (500 mL each) to obtain six fractions, SF-7343V-1 to SF-7343V-6. Compound **2** (9.0 mg) was purified from 30 mg of fraction SF-7343V-3 by C_18_ prep HPLC with the elution of a gradient solvent system of 30–100% MeOH in H_2_O with 0.01% of acid, over 40 min (t_R_ = 27 min). Fraction SF-7343V-4 (1.6249 g) was then subjected to a silica gel column (32 × 3 cm) eluted with CH_2_Cl_2_-MeOH (100:1–0:100) to obtain 11 sub-fractions (SF-7343V-4.1 to SF-7343V-4.11). Among these sub-fractions, sub-fraction SF7343-4.8 (280.8 mg) was further separated by a silica gel column (20 × 2 cm) eluted with CH_2_Cl_2_-MeOH (125:1) to provide sub-fractions SF7343-4.8.1 to SF7343-4.8.5. Sub-fraction SF7343-4.8.5 (49.5 mg) was subjected to C_18_ prep HPLC (50–75% methanol in H_2_O over 30 min) to obtain compound **4** (3.2 mg, t_R_ = 18.2 min). Finally, sub-fraction SF-7343V-4.9 (45.0 mg) was purified by C_18_ prep HPLC (eluted with a gradient solvent system of 50–100% MeOH in H_2_O, over 30 min) to obtain compounds **1** (4.2 mg, t_R_ = 19 min) and **3** (6.4 mg, t_R_ = 22 min).

#### 4.3.1. Compound **1**

Brown amorphous powder; HR-ESI-MS *m/z*: 327.0848 [M + Na]^+^ (calcd. for [C_16_H_16_O_6_Na]^+^, 327.0839). ^1^H NMR (DMSO-*d*_6_, 400 MHz): 10.22 (s, OH-3), 8.73 (s, OH-11), 8.70 (s, OH-10), 6.55 (s, H-12), 6.42 (d, *J* = 2.4 Hz, H-4), 6.40 (s, H-9), 6.13 (d, *J* = 2.4 Hz, H-6), 3.72 (s, OCH_3_-5), 3.41 (s, OCH_3_-1), 1.87 (s, H-14); ^13^C NMR (DMSO-*d*_6_, 100 MHz): 168.4 (C-1), 160.8 (C-5), 157.4 (C-3), 144.2 (C-10), 143.0 (C-7), 142.2 (C-11), 130.9 (C-8), 125.2 (C-13), 116.9 (C-12), 116.1 (C-9), 113.0 (C-2), 107.3 (C-6), 99.7 (C-4), 55.2 (O*C*H_3_-5), 51.4 (OCH_3_-1), 18.9 (C-14).

#### 4.3.2. Compound **2**

Yellowish powder; HR-ESI-MS *m/z*: 291.0878 [M + H]^+^ (calcd. for [C_15_H_15_O_6_]^+^, 291.0863). ^1^H NMR (DMSO-*d*_6_, 400 MHz): 8.67 (brs, OH-11), 8.63 (brs, OH-10), 6.54, (s, H-12), 6.43 (d, *J* = 2.7 Hz, H-4), 6.42 (s, H-9), 6.10 (d, *J* = 2.7 Hz, H-6), 3.76 (s, OCH_3_-5), 1.86 (s, H-14); ^13^C NMR (DMSO-*d*_6_, 100 MHz): 171.6 (C-1), 162.0 (C-5), 161.5 (C-3), 145.0 (C-7), 144.0 (C-10), 142.2 (C-11), 132.4 (C-8), 125.0 (C-13), 116.7 (C-12), 116.0 (C-9), 108.9 (C-6), 108.8 (C-2), 99.6 (C-4), 55.3 (OCH_3_-5), 18.9 (C-14).

#### 4.3.3. Compound **3**

White powder; HR-ESI-MS *m/z*: 259.0606 [M + H]^+^ (calcd. for [C_14_H_11_O_5_]^+^, 259.0601); 281.0428 [M + Na]^+^ (Calcd. for [C_14_H_10_O_5_Na]^+^, 281.0420). ^1^H NMR (DMSO-*d*_6_, 400 MHz): 7.22 (d, *J* = 2.0 Hz, H-6), 6.70 (d, *J* = 2.7 Hz, H-12), 6.62 (d, *J* = 2.7 Hz, H-10), 6.35 (d, *J* = 2.0 Hz, H-4), 2.68 (s, H-14); ^13^C NMR (DMSO-*d*_6_, 100 MHz): 165.6 (C-5), 164.7 (C-1), 164.1 (C-3), 158.4 (C-11), 152.6 (C-9), 138.3 (C-13), 138.1 (C-7), 117.5 (C-12), 109.0 (C-8), 104.4 (C-6), 101.6 (C-10), 100.9 (C-4), 97.2 (C-2), 25.2 (C-14).

#### 4.3.4. Compound **4**

Brown amorphous powder; [*α*]^25^_D_ −25.5 (*c* 0.22, MeOH). HR-ESI-MS *m/z*: 293.1028 [M + H]^+^ (calcd. for [C_15_H_17_O_6_]^+^, 293.1020); 315.0852 [M + Na]^+^ (Calcd. for [C_15_H_16_O_6_Na]^+^, 315.0839). ^1^H NMR (DMSO-*d*_6_, 400 MHz): 11.28 (s, OH-3), 6.75 (d, *J* = 2.3 Hz, H-6), 6.51 (d, *J* = 2.3 Hz, H-4), 6.30 (d, *J* = 3.2 Hz, H-6′), 5.31 (d, *J* = 5.6 Hz, OH-5′), 5.13 (brs, OH-4′), 3.95 (m, H-5′), 3.86 (s, OCH_3_-5), 3.70 (m, H-4′), 2.26 (dd, *J* = 3.2, 14.0 Hz, H_e_-3′), 1.95 (dd, *J* = 7.2, 14.0 Hz, H_a_-3′), 1.47 (s, H-7′).

### 4.4. Cell Culture and Reagents

HaCaT cells were donated by Hyeonsook Cheong, Chosun University (Kwangju, Korea), and the cells were cultured in DMEM with 10% FBS (Gibco, New york, NY, USA), and tin protoporphyrin (Snpp; Calbiochem, Merck, Darmstadt, Germany). Recombinant human TNF-α and IFN-γ, and ELISA kits for IL-8, IL-6, and RANTES were obtained from BioLegend (San Diego, CA, USA). Antibodies against ICAM-1, FLG, IVL, actin, PCNA, HRP-conjugated anti-mouse, and anti-rabbit IgG were purchased from Santa Cruz Biotechnology (Santa Cruz, CA, USA). Antibodies against p-IκBα, IκBα, p65, p-stat1, p-stat3, HO-1, and Nrf2 were purchased from Cell Signaling Technology (Danvers, MA, USA). Other chemical reagents were purchased from Sigma-Aldrich Chemical Company (St. Louis, MO, USA).

### 4.5. MTT Assay

To determine cell viability, cells were maintained at a density of 2 × 104 cells in 48-well plates and then treated with compound **1** (20–80 μM), compound **2** (5–40 μM), compound **3** (12.5–20 μM) and compound **4** (10–80 μM). After incubation for 24 h, the cell culture medium was removed from each well and replaced with 500 μL of fresh medium in each well. The cells were incubated with 0.5 mg/mL of 3-(4,5-dimethylthiazol-2-yl)-2,5-diphenyltetrazolium bromide (MTT) for 1 h, and the formed formazan was dissolved in dimethyl sulfoxide (DMSO, St. Louis, MO, USA). The absorbance of the dissolved formazan was measured at a wavelength of 540 nm by using an ELISA microplate reader (Molecular Devices, San Jose, CA, USA).

### 4.6. Measurement of Cytokines and Chemokines

HaCaT cells (5.0 × 10^6^ cells/mL) were seeded in a 12-well plate. For ELISA analysis, the cells were pre-treated with compounds for 3 h and then stimulated with TNF-α/IFN-γ (each for 5 ng/mL), with co-treatment for 24 h. After that, the cell culture supernatant was collected and IL-6, IL-8, RANTES, and MDC levels were evaluated using an ELISA kit, according to the manufacturer’s instructions. For experiments of compound **1** with Snpp, firstly, HaCaT cells were treated with Snpp (40 μM) for 2 h, and then the compound **1** was added into the culture media for another 3 h, Finally, we stimulated TNF-α/IFN-γ with HaCaT cell for 24 h. After that, the cell culture supernatant was collected and IL-6, IL-8, and MDC were detected using an ELISA kit (Biolegend, San Diego, CA, USA).

### 4.7. Extraction of Total, Nuclear, and Cytosolic Protein

For total protein, the cells were scraped, harvested, and lysed with RIPA buffer (Thermo Fisher Scientific, MA, USA). Nuclear and cytoplasmic protein fractions were extracted using a Nuclear Extraction Kit (Cayman Chemical, Ann Arbor, MI, USA), according to the manufacturer’s instructions. All proteins were stored at −80 °C until use. HaCaT cells were pre-treated with indicated concentrations of compound **1** and **3**. For Western blot analysis of ICAM-1, FLG, and IVL, the cells were induced with TNF-α/IFN-γ (each for 5 ng/mL) for 24 h. For analysis of p-IκBα, IκBα, p65, p-stat1, and p-stat3, the cells were induced with TNF-α/IFN-γ for 15 min.

### 4.8. Western Blot Analysis

Proteins were separated by electrophoresis on an SDS-PAGE gel and transferred to nitrocellulose membranes. The membrane was blocked with 5% skim milk (dissolved in TBST) for 45–60 min at room temperature. They were then incubated overnight at 4 °C with primary antibodies (1:1000 dilution). After washing thrice with TBST, the membranes were incubated with horseradish peroxidase-conjugated secondary antibody (1:5000 dilution) for 1 h at room temperature. After washing with TBST, specific proteins were detected using ECL solution. The membranes were analyzed using the ImageJ software (National Institutes of Health, Rockville, MD, USA).

### 4.9. NF-κB DNA Binding Assay

For experiments of compound **1** with Snpp, firstly, HaCaT cells were treated with Snpp (40 μM) for 2 h, and then we added the compound 1 into the culture media for another 3 h, Finally, we stimulated HaCaT cells with TNF-α/IFN-γ for 15 min. After that, the cell was collected, nuclear protein fractions were extracted using a Nuclear Extraction Kit.

NF-κB p65-DNA binding was detected using an NF-κB p65 Transcription Factor Assay Kit (10007889, Cayman Chemical). The nuclear fraction was used to quantify the relative nuclear NF-κB p65-DNA binding activity. This was evaluated in accordance with the manufacturer’s instructions.

### 4.10. Statistical Analysis

The result values for each group were represented as the mean ± standard deviation (SD), and the GraphPad Software (San Diego, CA, USA) was used to carry out one-way analysis of variance (ANOVA), followed by Duncan’s multiple comparison tests to test the significance. Statistical significance was set at ^#^ *p* < 0.05, ^##^ *p* < 0.01, ^###^ *p* < 0.001 vs. control. * *p* < 0.05, ** *p* < 0.01, *** *p* < 0.001 vs. TNF-α/IFN-γ-treated group. ^△^ *p* < 0.05, ^△△^ *p* < 0.01, ^△△△^ *p* < 0.001 vs. compound **1**+TNF-α/IFN-γ-treated group

## 5. Conclusions

In this study, the effects of 4 compounds isolated from fungal strain SF-7343 metabolites on skin inflammation were examined. Alternate C (**1**) and alternariol (**3**) had inhibitory effects on IL-6, IL-8, MDC, and RANTES secretion; the expression of ICAM-1 was also decreased. Furthermore, alternate C (**1**) and alternariol (**3**) also upregulated the expression of FLG and IVL. Further mechanistic studies showed that alternate C (**1**) and alternariol (**3**) inhibited the nuclear translocation of NF-κB p65 and STAT1 and STAT3 activation. Alternate C (**1**) significantly induced the expression of HO-1, which exerts anti-inflammatory effects by inhibiting the secretion of IL-6, IL-8, MDC, and RANTES. These results demonstrate that the compounds obtained from the fungal strain SF-7343 metabolites could be further developed as preventive agents on the regulation of skin inflammation in human keratinocytes. These data may be used as a basis for the development of preventive materials strategies for atopic dermatitis.

## Figures and Tables

**Figure 1:**
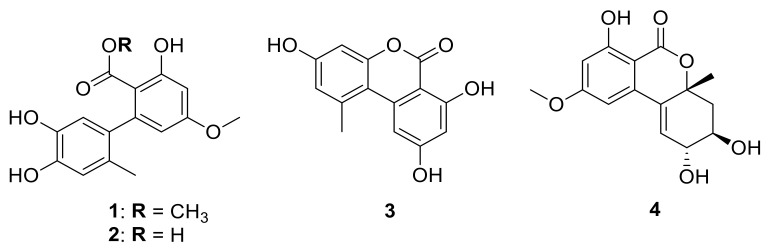
Structure of isolated compounds (**1**–**4**).

## Data Availability

The data presented in this study are available within the article. Other data that support the findings of this study are available upon request from the corresponding author.

## Supplementary Materials

The following are available online at https://www.mdpi.com/article/10.3390/ijms22189674/s1.
