# Supplementary material for: Anti-Inflammatory Effects of Metabolites from Antarctic Fungal Strain Pleosporales sp. SF-7343 in HaCaT Human Keratinocytes
Source: Int J Mol Sci. 2021 Sep 7;22(18):9674. doi: 10.3390/ijms22189674 (PMC8468741; doi:10.3390/ijms22189674)
Supplement: Supplementary file 1 [file ijms-22-09674-s001.zip › ijms-1354389-supplementary.pdf]

Supporting Information for

Figure S1. HREIMS of **1**

Figure S2. ^{1}H NMR spectrum (DMSO-d_6, 400 MHz) of **1**

Figure S3. ^{13}C NMR spectrum (DMSO-d_6, 100 MHz) of **1**

Figure S4. COSY spectrum of **1**

Figure S5. HMQC spectrum of **1**

Figure S6. HMBC spectrum of **1**

Figure S7. HREIMS of **2**

Figure S8. ^{1}H NMR spectrum (DMSO-d_6, 400 MHz) of **2**

Figure S9. ^{13}C NMR spectrum (DMSO-d_6, 100 MHz) of **2**

Figure S10. COSY spectrum of **2**

Figure S11. HMQC spectrum of **2**

Figure S12. HMBC spectrum of **2**

Figure S13. HREIMS of **3**

Figure S14. ^{1}H NMR spectrum (DMSO-d_6, 400 MHz) of **3**

Figure S15. ^{13}C NMR spectrum (DMSO-d_6, 100 MHz) of **3**

Figure S16. COSY spectrum of **3**

Figure S17. HMQC spectrum of **3**

Figure S18. HMBC spectrum of **3**

Figure S19. HREIMS of **4**

Figure S20. ^{1}H NMR spectrum (DMSO-d_6, 400 MHz) of **4**

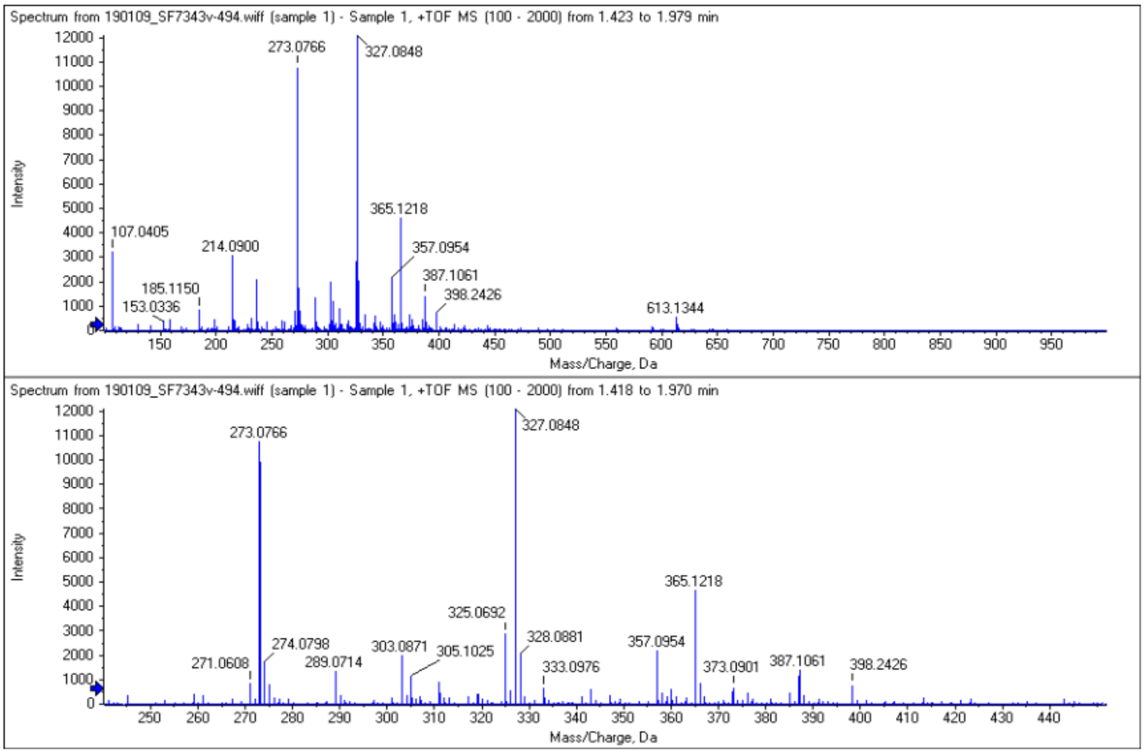

Figure S1. HREIMS of **1**

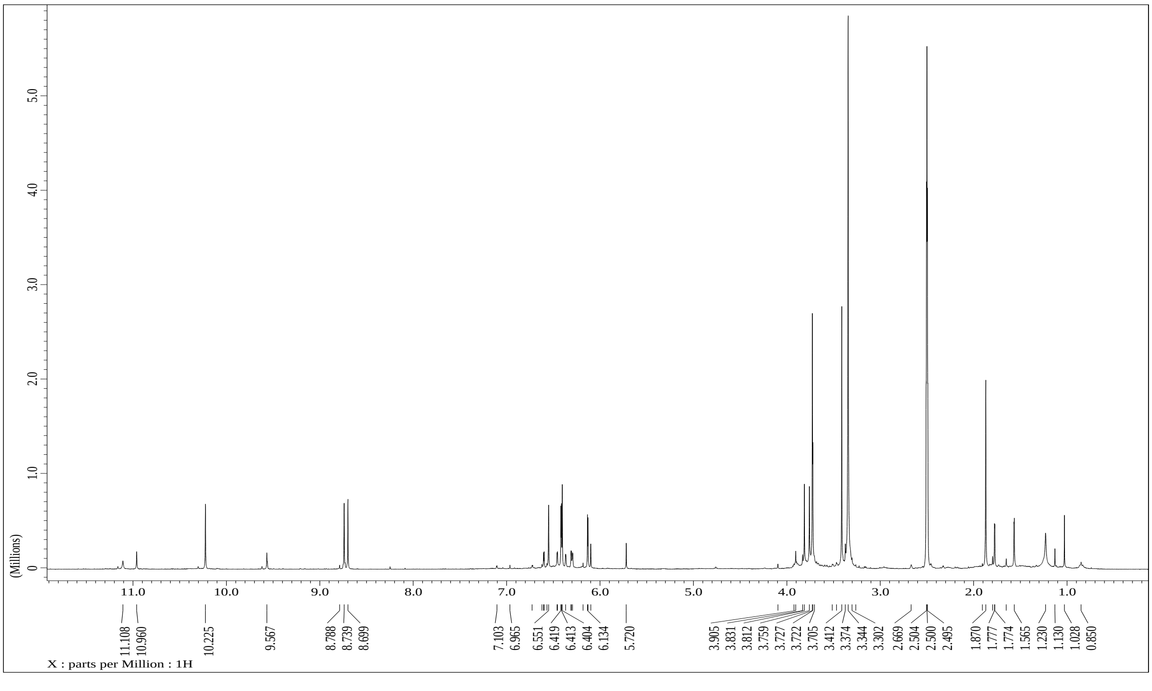

Figure S2. ^{1}H NMR spectrum (DMSO-d_6, 400 MHz) of **1**

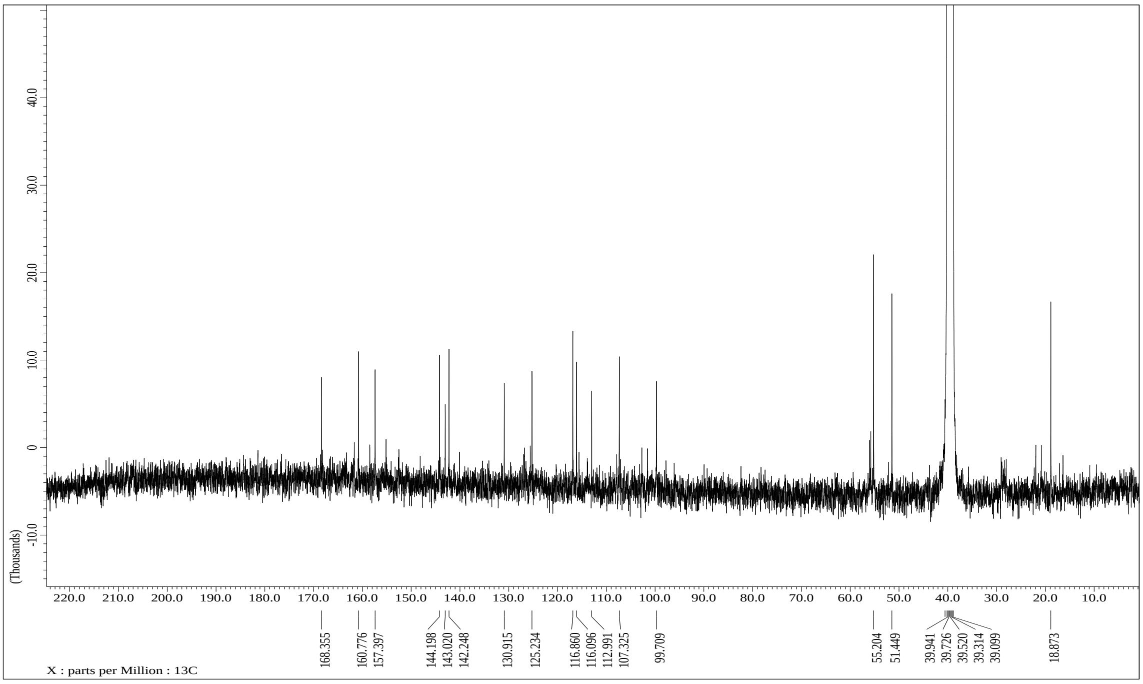

Figure S3. ^{13}C NMR spectrum (DMSO-d_6, 100 MHz) of **1**

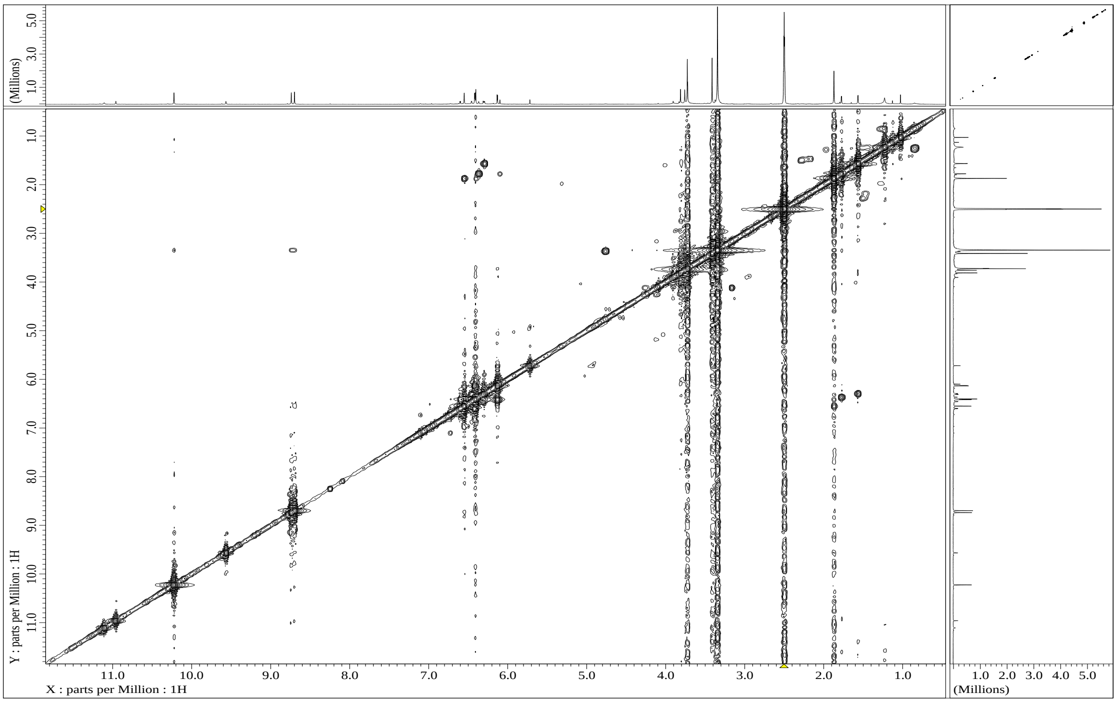

Figure S4. COSY spectrum of **1**

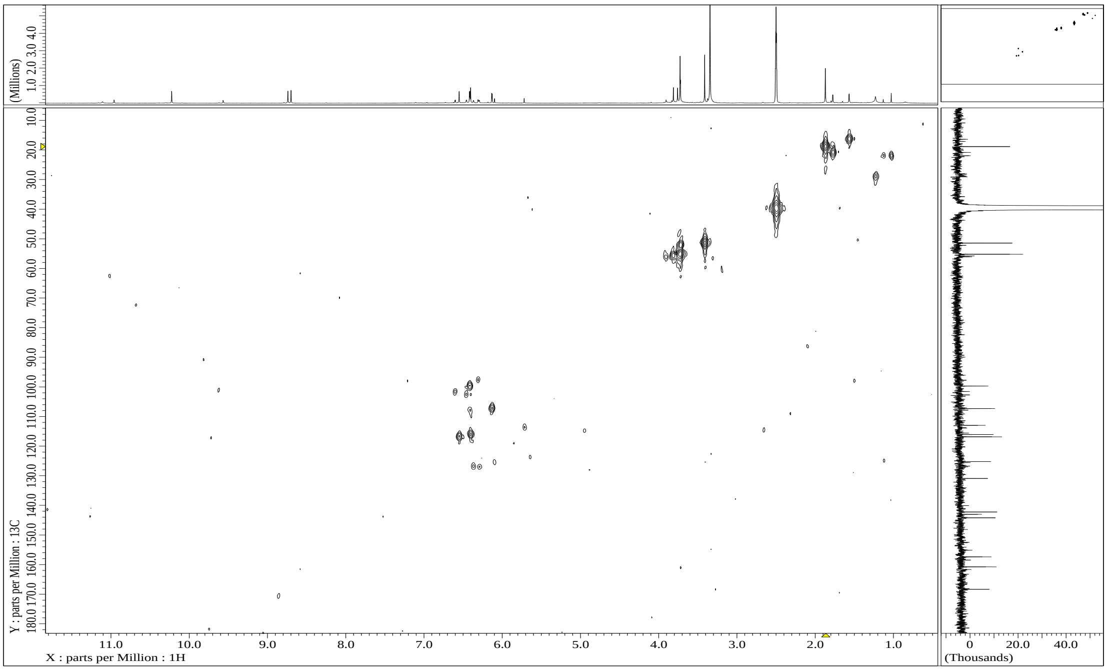

Figure S5. HMQC spectrum of **1**

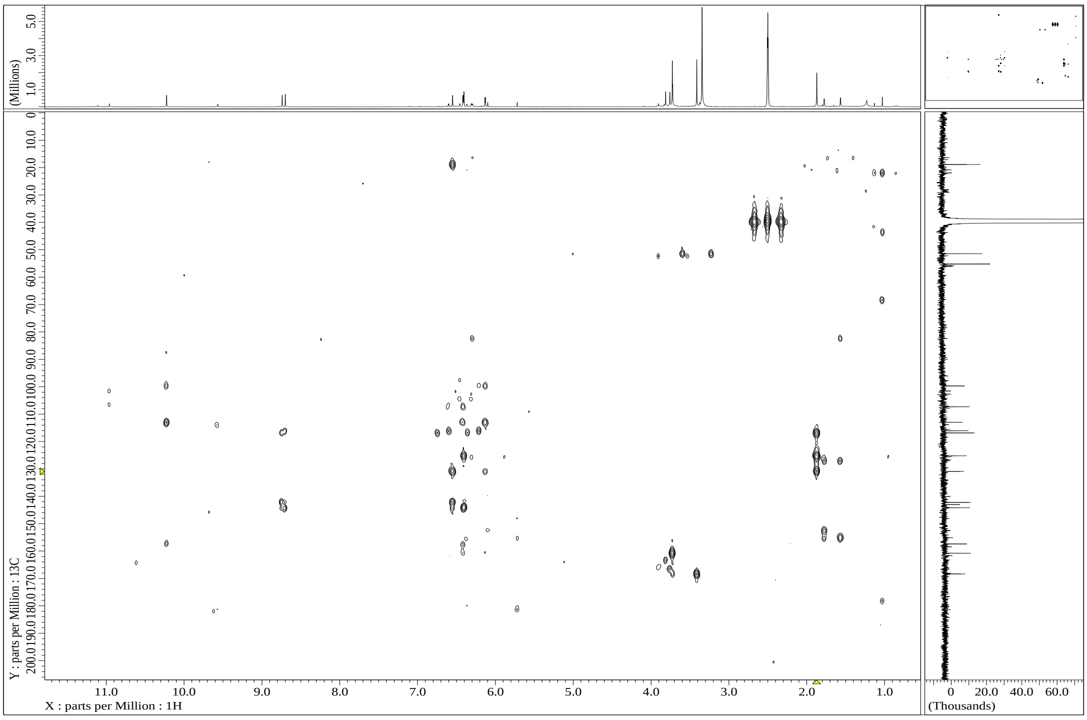

Figure S6. HMBC spectrum of **1**

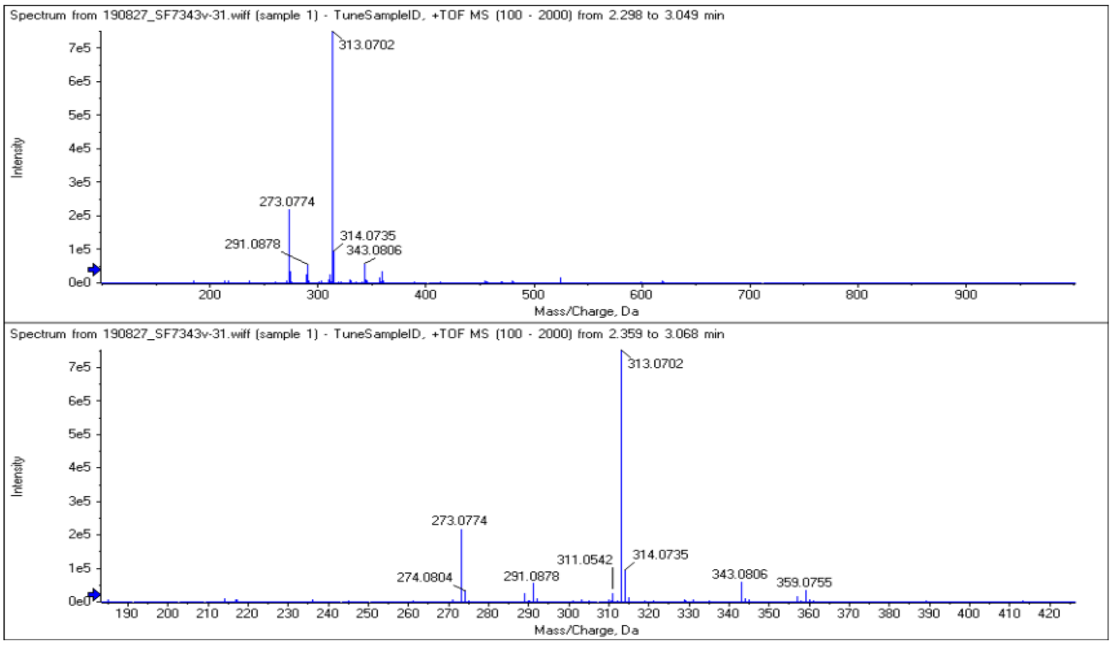

Figure S7. HREIMS of **2**

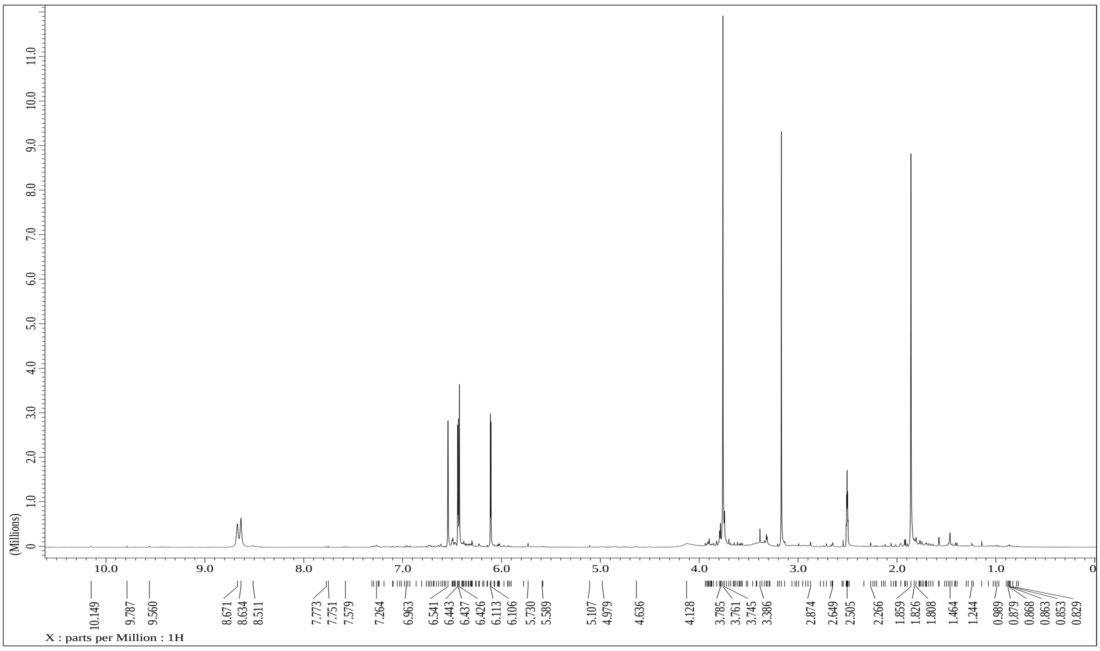

Figure S8. ^{1}H NMR spectrum (DMSO-d_6, 400 MHz) of **2**

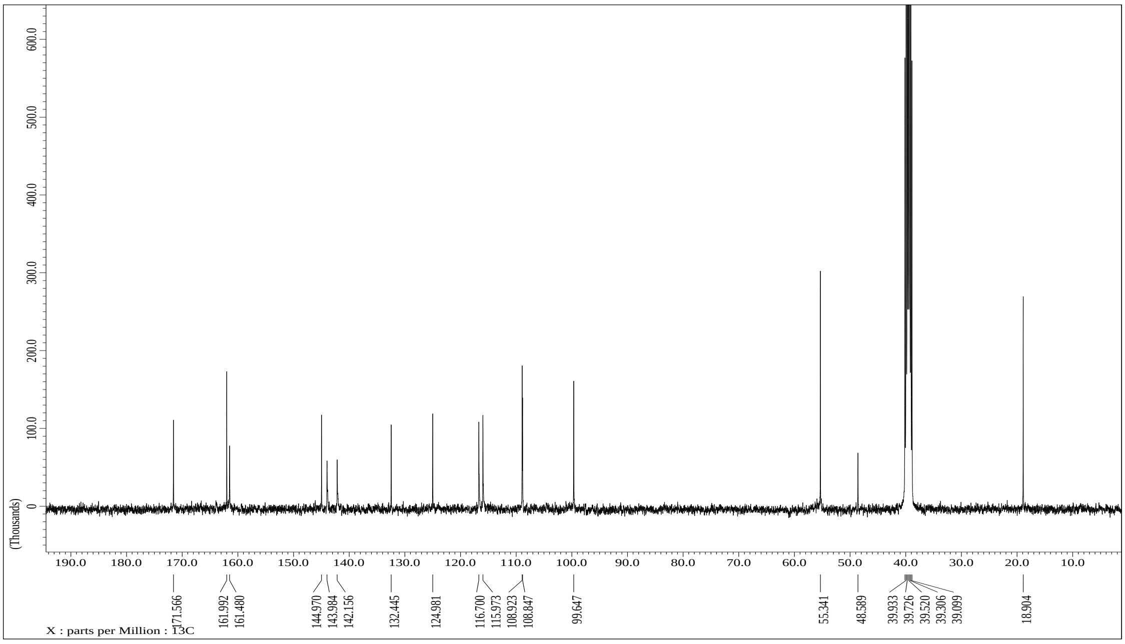

Figure S9. ^{13}C NMR spectrum (DMSO-d_6, 100 MHz) of **2**

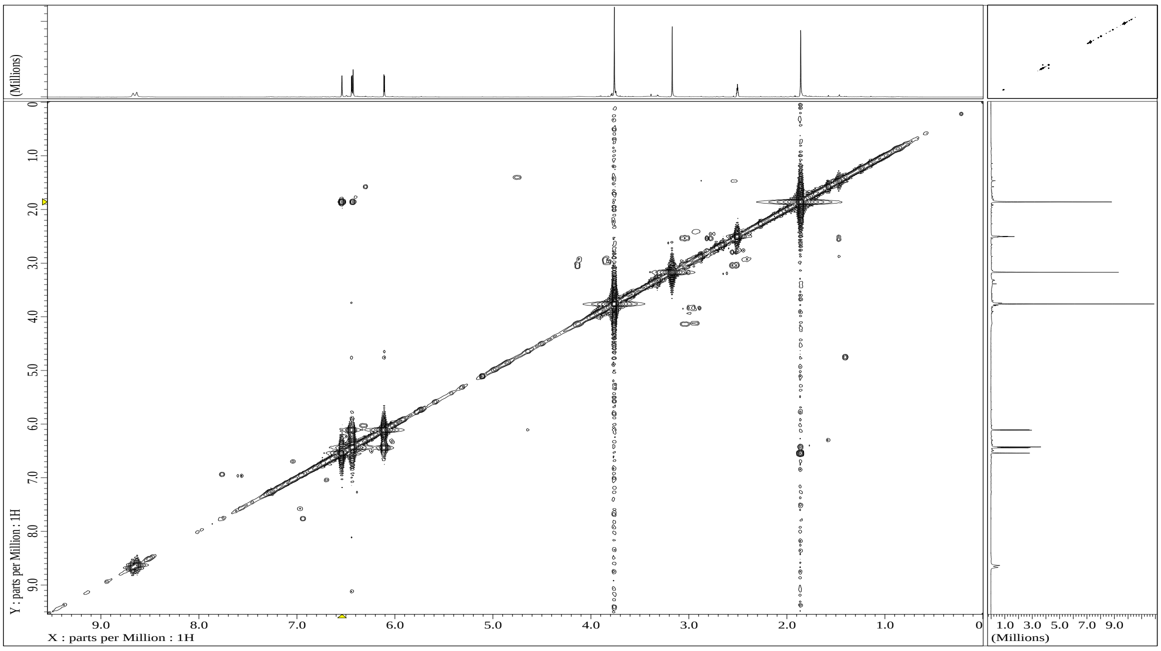

Figure S10. COSY spectrum of **2**

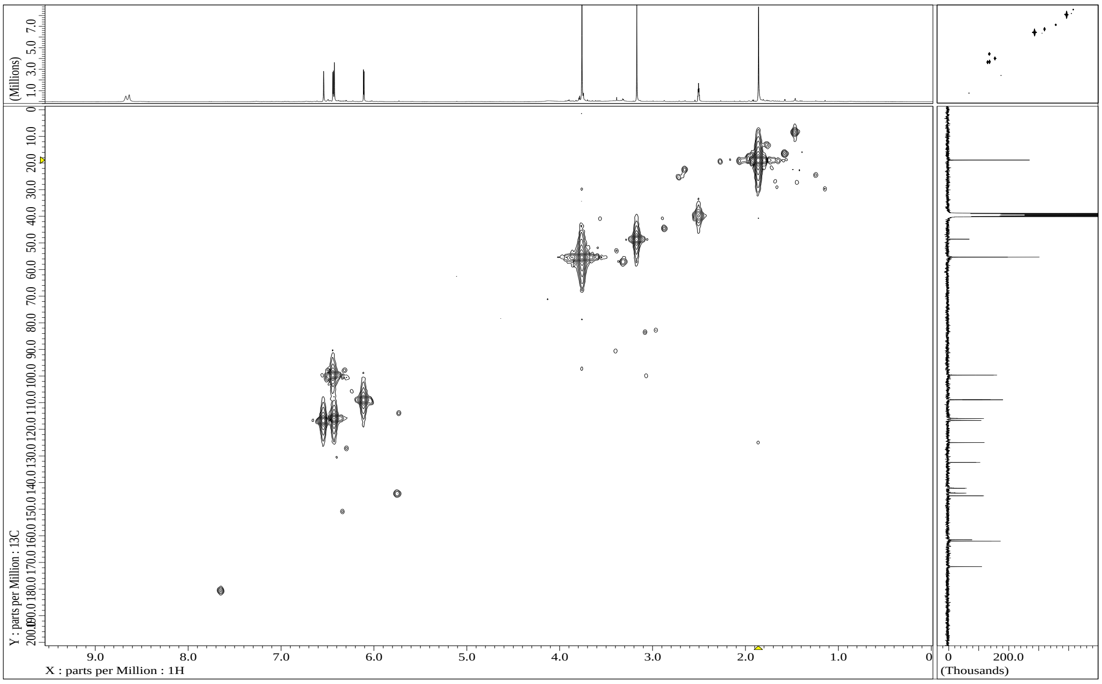

Figure S11. HMQC spectrum of **2**

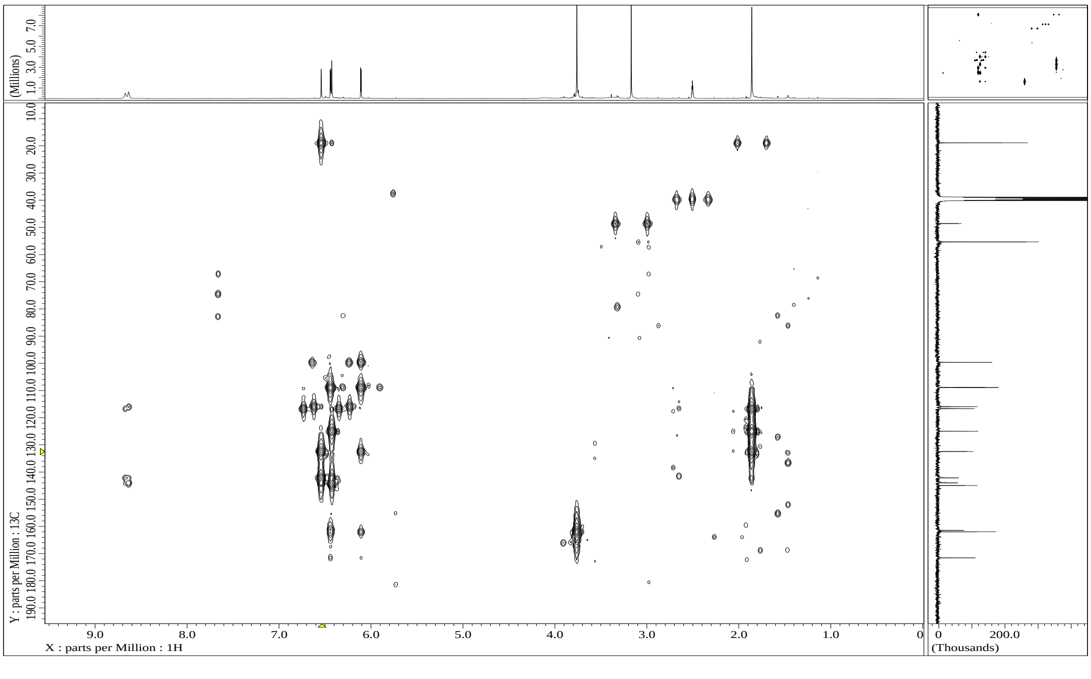

Figure S12. HMBC spectrum of **2**

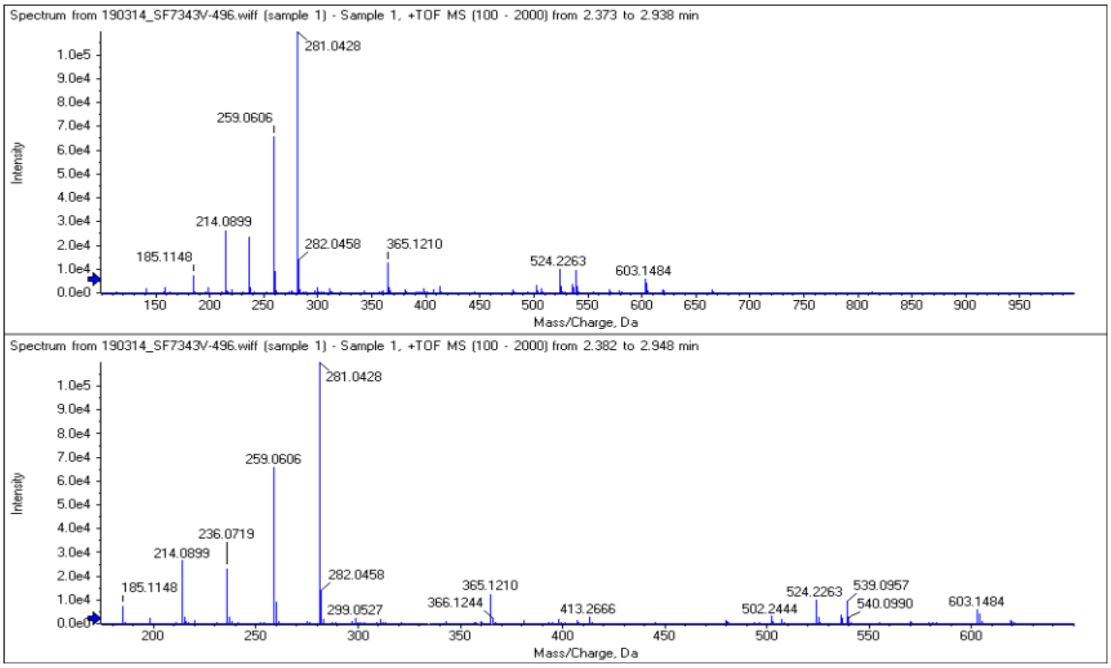

Figure S13. HREIMS of **3**

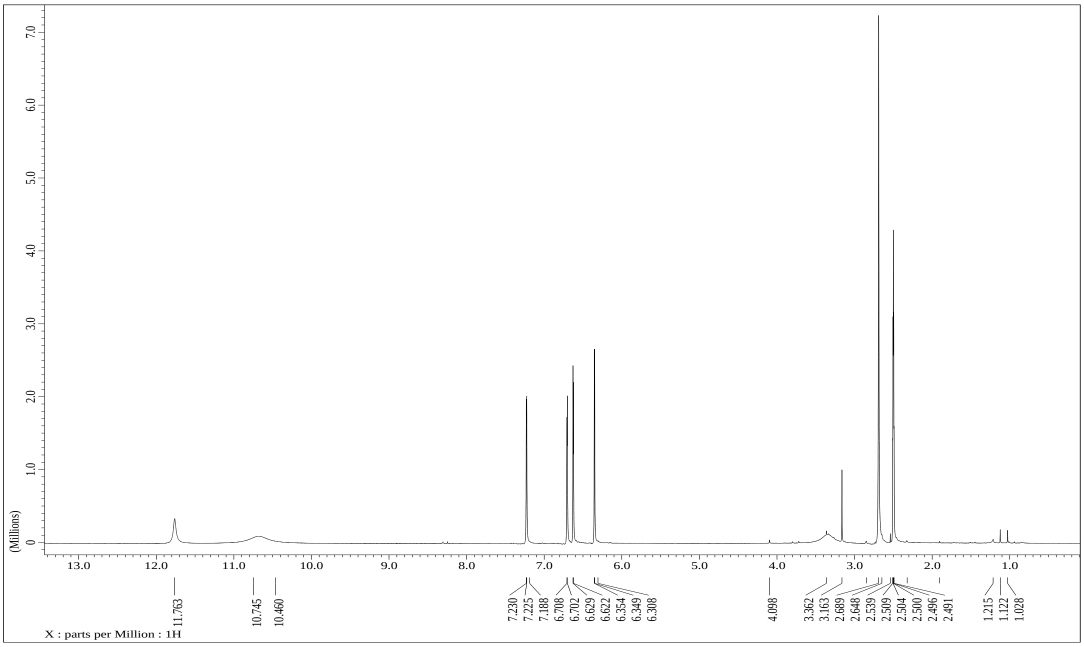

Figure S14. ^{1}H NMR spectrum (DMSO-d_6, 400 MHz) of **3**

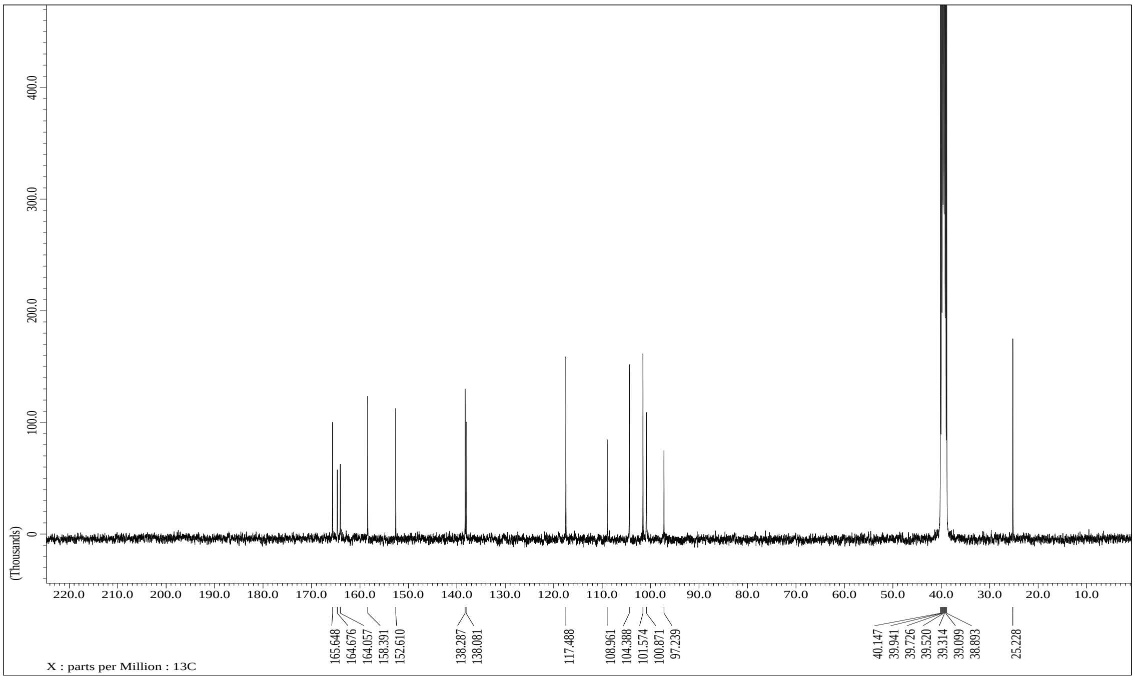

Figure S15. ^{13}C NMR spectrum (DMSO-d_6, 100 MHz) of **3**

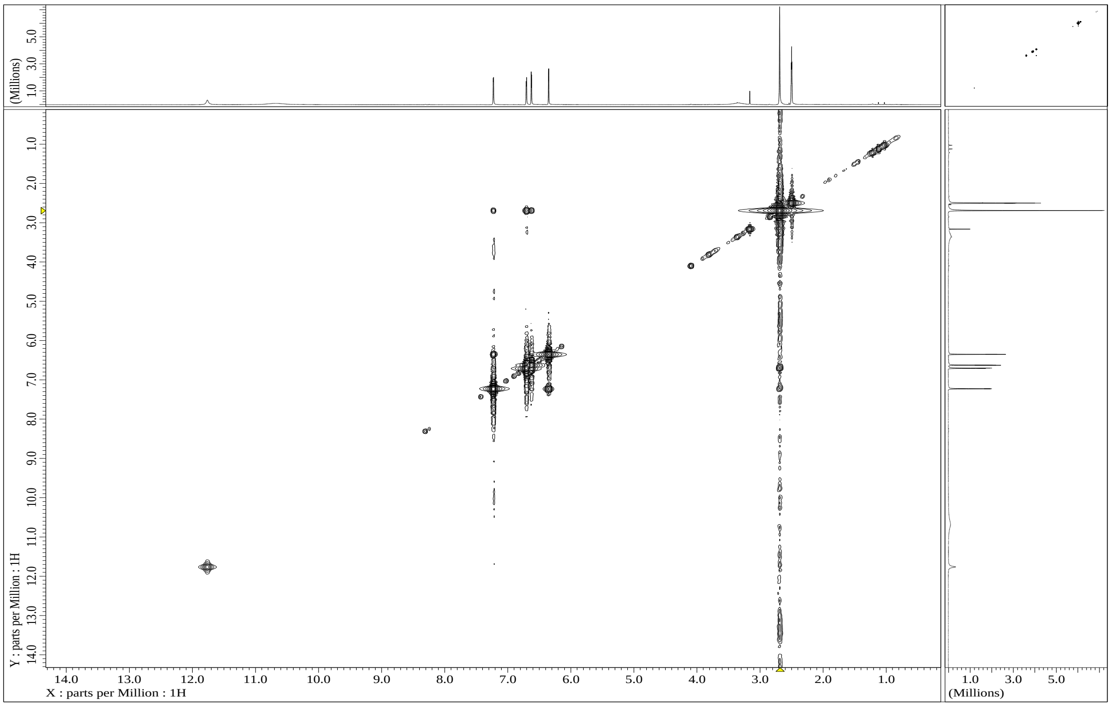

Figure S16. COSY spectrum of **3**

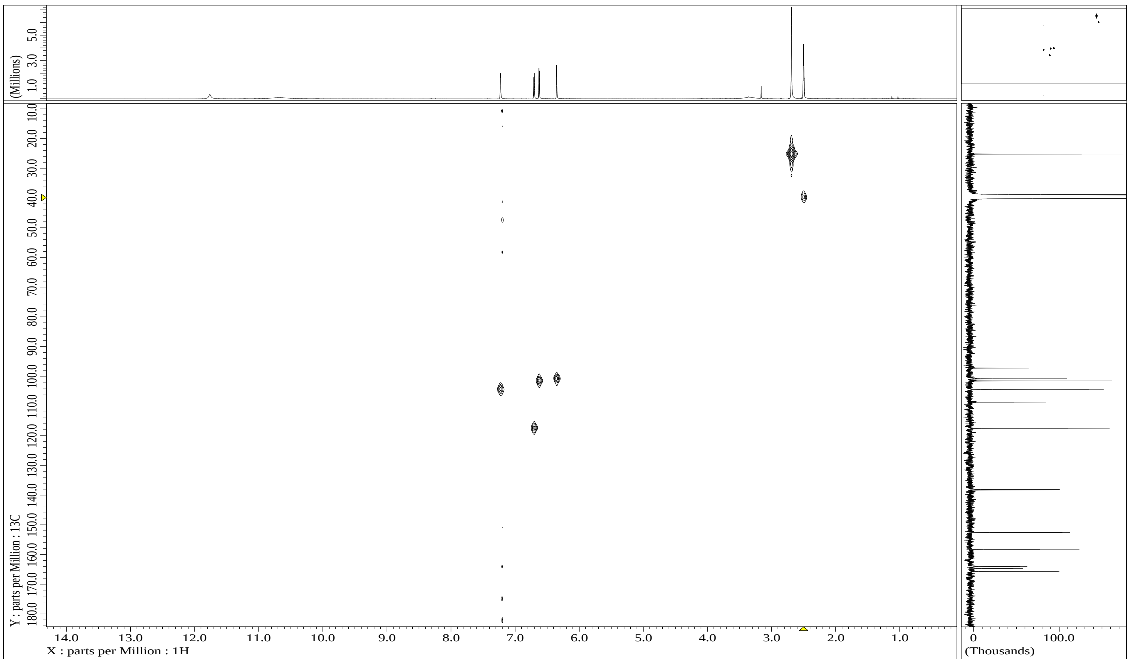

Figure S17. HMQC spectrum of **3**

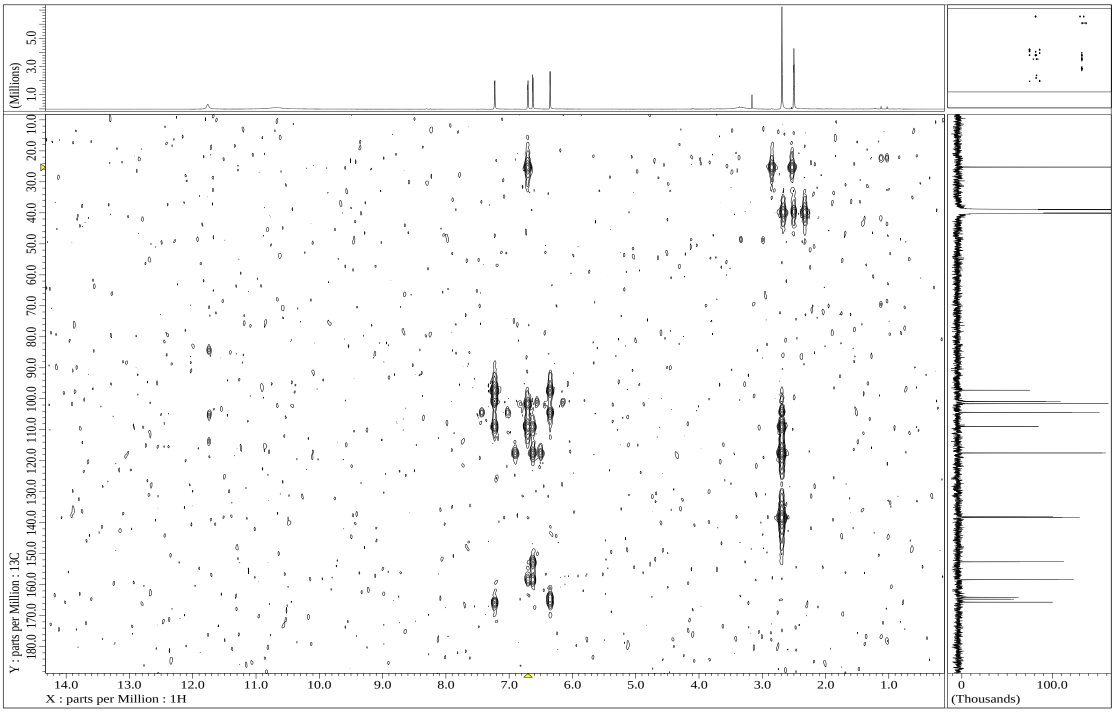

Figure S18. HMBC spectrum of **3**

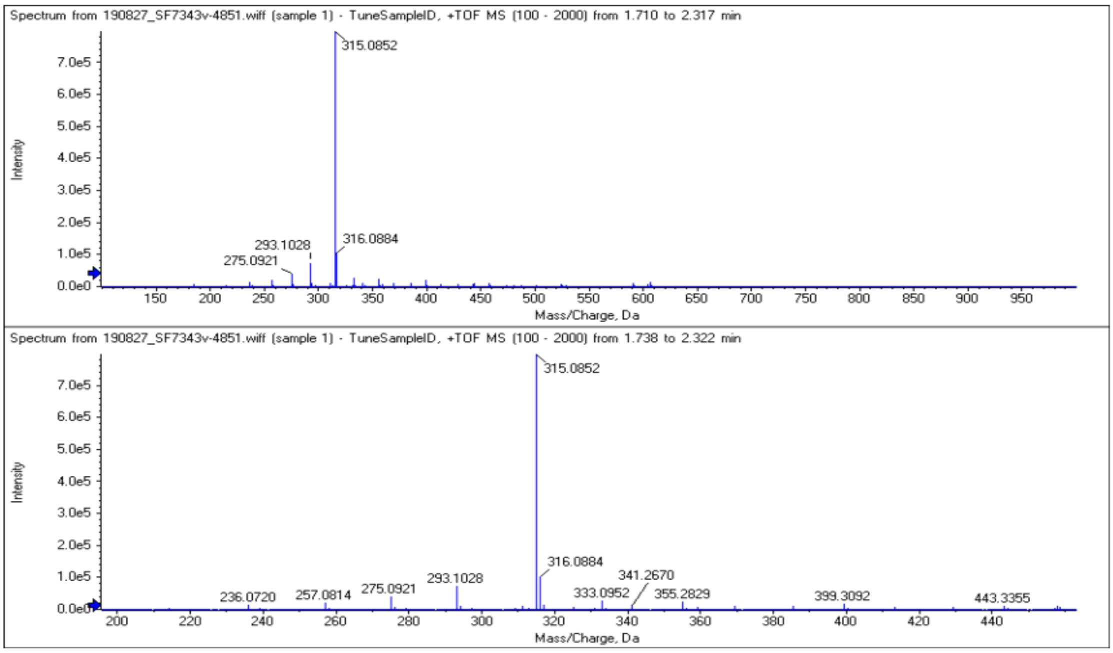

Figure S19. HREIMS of **4**

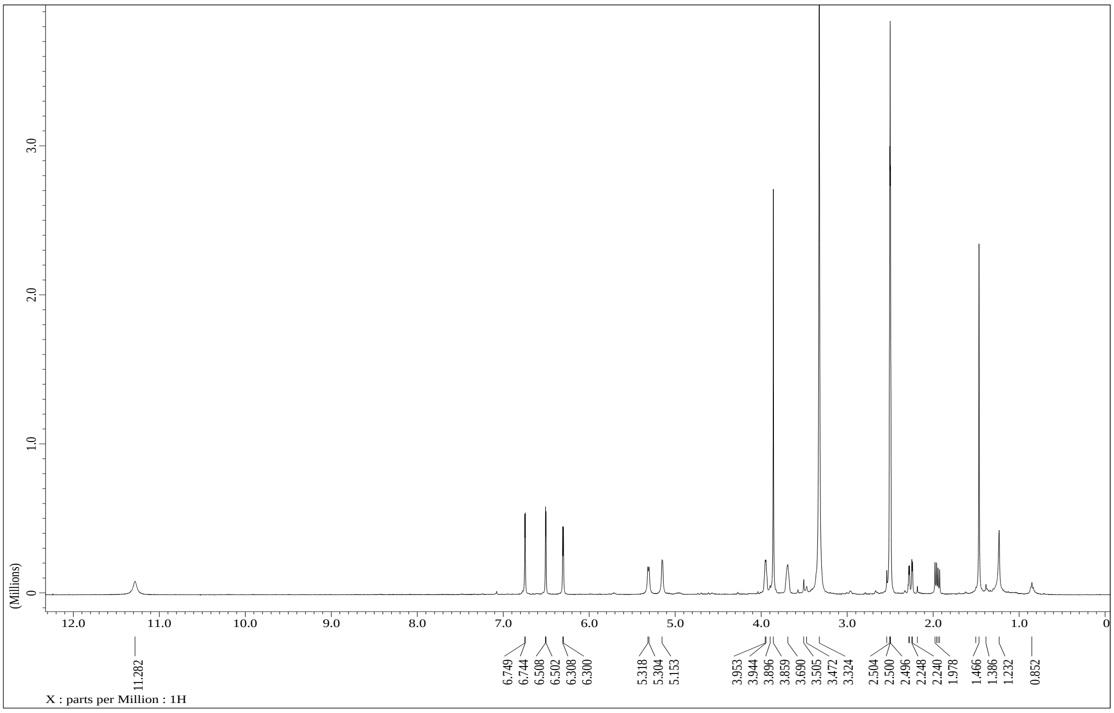

Figure S20. ^{1}H NMR spectrum (DMSO-d_6, 400 MHz) of **4**
